# Supplementary material for: Vibration Induced Transport of Enclosed Droplets
Source: Micromachines (Basel). 2019 Jan 19;10(1):69. doi: 10.3390/mi10010069 (PMC6356547; doi:10.3390/mi10010069)
Supplement: Supplementary file 1 [file micromachines-10-00069-s001.zip › Vibration Induced Transport of Enclosed Droplets_SUPPLEMENTARY_micromachines.docx]

Supplementary

Vibration Induced Transport of Enclosed Droplets

Hal R. Holmes^1^, and Karl F. Böhringer^1,2,*^

^1^ Department of Bioengineering, University of Washington, Seattle, WA USA 98105

^2^ Department of Electrical Engineering, University of Washington, Seattle, WA USA 98195

***** Correspondence: [karlb@uw.edu](mailto:karlb@uw.edu)


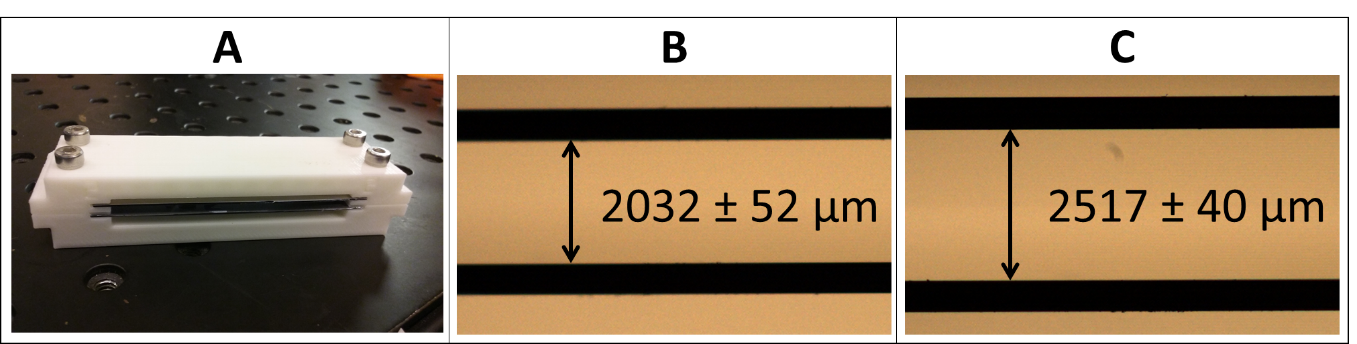


Figure S1: Dual-plate set up. A 3D printed fixture was fabricated to enclose droplets (A). The dimensions of the fixture were optimized to provide a 2.0 mm (B) and 2.5 mm (C) separation between the two plates. The tolerance of this set up was measured to be within 3% of the target dimension for 2.0 mm and within 2% of the target dimension for 2.5 mm separations.

© 2018 by the authors. Submitted for possible open access publication under the terms and conditions of the Creative Commons Attribution (CC BY) license (http://creativecommons.org/licenses/by/4.0/).
